# Screening of Bioactive Properties in Brown Algae from the Northwest Iberian Peninsula

**DOI:** 10.3390/foods10081915

**Published:** 2021-08-18

**Authors:** Aurora Silva, Carla Rodrigues, Paula Garcia-Oliveira, Catarina Lourenço-Lopes, Sofia A. Silva, Pascual Garcia-Perez, Ana P. Carvalho, Valentina F. Domingues, M. Fátima Barroso, Cristina Delerue-Matos, Jesus Simal-Gandara, Miguel A. Prieto

**Affiliations:** 1 Nutrition and Bromatology Group, Department of Analytical and Food Chemistry, Faculty of Food Science and Technology, University of Vigo, Ourense Campus, E32004 Ourense, Spain; mass@isep.ipp.pt (A.S.); paula.garcia.oliveira@uvigo.es (P.G.-O.); c.lopes@uvigo.es (C.L.-L.); pasgarcia@uvigo.es (P.G.-P.); mprieto@uvigo.es (M.A.P.); 2 REQUIMTE/LAQV, Instituto Superior de Engenharia do Instituto Politécnico do Porto, Rua Dr António Bernardino de Almeida 431, 4200-072 Porto, Portugal; 1190133@isep.ipp.pt (C.R.); vfd@isep.ipp.pt (V.F.D.); cmm@isep.ipp.pt (C.D.-M.); 3 Departamento de Química, Universidade de Aveiro, 3810-193 Aveiro, Portugal; sofia.silva96@gmail.com (S.A.S.); apcarvalho@ucp.pt (A.P.C.); 4 CBQF—Centro de Biotecnologia e Química Fina—Laboratório Associado, Escola Superior de Biotecnologia, Universidade Católica Portuguesa, Rua Diogo Botelho 1327, 4169-005 Porto, Portugal

**Keywords:** macroalgae, brown algae, phenolic content, antioxidants, bioactive compounds, antimicrobial activity

## Abstract

Algae are an underexploited source of natural bioactive compounds in Western countries, so an increasing interest in the valorization of these marine organisms has emerged in recent years. In this work, the effect of extracting solvent on the extraction yield, phenolic content, antioxidant capacity, and antimicrobial activity of nine brown macroalgae species (*Ascophyllum nodosum*, *Himanthalia elongata*, *Undaria pinnatifida*, *Pelvetia canaliculata*, *Saccharina latissima*, *Bifurcaria bifurcata*, *Laminaria ochroleuca*, *Sargassum muticum*, and *Fucus spiralis*) was assessed. Total phenolic content (TPC) and the antioxidant properties of extracts by different assays: radical scavenging activity (DPPH-RSA) and ferric reducing antioxidant power (FRAP) were performed. The antimicrobial activity of extracts was studied against six different foodborne microorganisms: *Staphylococcus aureus*, *Staphylococcus epidermidis*, *Bacillus cereus*, *Escherichia coli*, *Salmonella enteritidis*, and *Pseudomonas aeruginosa.* The highest extraction yield was achieved in ethanolic extracts. However, the highest TPC and FRAP values were obtained on the ethyl acetate extracts, especially from *A. nodosum*. Concerning algal species, the highest TPC and FRAP values were found in *A. nodosum*, while the highest DPPH-RSA values were achieved in the hexane extracts of *B. bifurcata*. The antimicrobial activity of algal extracts varied according to the solvent and alga selected, suggesting the species- and solvent-dependent behavior of this property, with *B. bifurcata* extracts showing the highest results for a wide range of bacteria. Our results provide insight on the characterization of widespread brown algae in the coasts of the North-Western region of the Iberian Peninsula, reflecting multiple health-enhancing properties which may lead to their exploitation in food, pharmacological, and cosmetic industries.

## 1. Introduction

**Figure 1:**
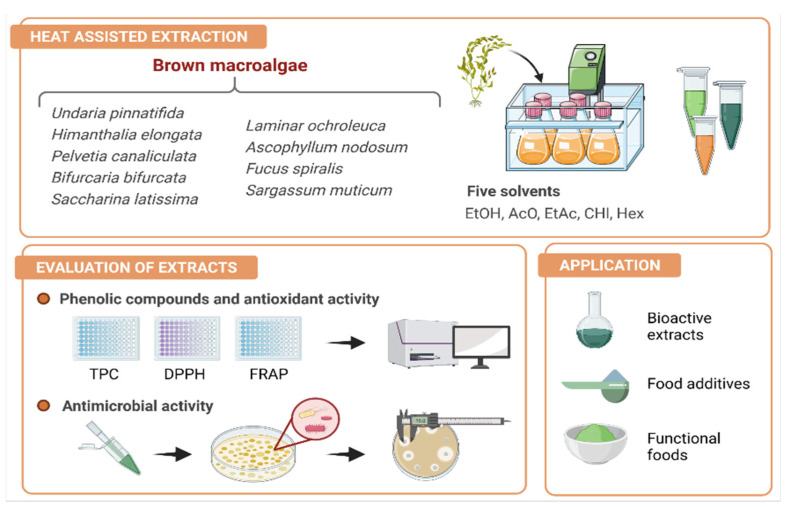
Schematic representation of the main goals of the present study. The nine selected species were extracted by heat-assisted extraction using five different solvents and the obtained extracts were evaluated in terms of phenolic content and antioxidant and antimicrobial properties. These bioactive extracts could have different applications such as food additives of functional foods.

The topic of food safety is one of the most widespread concerns, as the European Food Safety Authority and the European Centre for Disease Prevention and Control reported the occurrence of 91,662 confirmed cases of disease just from salmonellosis in Europe [1]. Some foodborne microorganisms, for instance *Bacillus cereus*, can be found in different matrices, like soil and plants, being able to thrive in the intestinal tract of animals and further cause major health problems [2]. Consequently, the control of pathogenic microorganisms in food products is a major issue for this industry. Recently, consumer pressure and environmental awareness have led to a trend to opting for more natural ingredients. Thus, marine algae can be considered as functional foods and ingredients of natural origin, since they are used in food and cosmetic products, as well as in traditional remedies in Asian countries. Nevertheless, macroalgae are still underestimated in Western cultures, despite the numerous scientific studies that have proved their biological activities (Figure 1). Such health-promoting properties associated with macroalgae have prompted their use in various new industrial applications, also motivated by their chemical and nutritional composition and their high availability in coastal ecosystems [3,4].

Among the different groups of macroalgae, brown algae have gained attention due to the numerous biological properties and bioactive compounds that have been attributed to these organisms [5,6,7]. *Himanthalia elongata* (L.) S. F. Gray, *Undaria pinnatifida* (Harvey) Suringar, 1873, *Pelvetia canaliculata* (L.) Decne. and Thur., *Laminaria ochroleuca* Bach. Pyl., *Saccharina latissima* L., *Bifurcaria bifurcata* R. Ross, 1958, *Fucus spiralis* L., and *Ascophyllum nodosum* (L.) Le Jolis and the invasive species *Sargassum muticum* (Yendo) Fensholt are brown macroalgae species that can be found in the Northwestern coasts of the Iberian Peninsula. Besides their widespread distribution, these species were widely reported for their high nutritional value and associated beneficial properties to human health [8]. Some activities that have been recognized to brown macroalgae include antioxidant [5,9], anti-inflammatory [10,11], or antimicrobial [12,13], among others. Several studies have reported that the presence of phenolic compounds is linked to the biological properties attributed to these organisms, as it is the case of the antioxidant, antimicrobial, and cytotoxic activities [5,11,14]. Nevertheless, the chemical composition of macroalgae presents great variations depending on different factors, like species, geographical region, seasonal variations, and other environmental factors [15,16,17]. Despite these health-promoting properties, macroalgae are still considered as underexploited resources and greater efforts are needed to achieve their chemical and bioactive characterization, facing their large-scale application by different industrial sectors.

The chemical composition of macroalgae presents great variations depending on different factors, like species, geographical region, seasonal variations, and other environmental factors [15,16,17]. Thus, to achieve such goal, the development of efficient experimental procedures to maximize the extraction of bioactive compounds from brown macroalgae is of great interest for both the food and cosmetic industries, throughout the optimization of critical factors involved in this process, such as extraction method, solvent polarity, incubation time, etc. Among them, the chemical nature of the solvent used for extraction plays a fundamental role, as it should promote the solubility of target compounds and respond to other additional concerns, including safety and environmental features [18].

In this work, we investigated the influence of the extracting solvent on the recovery of phenolic compounds from different brown macroalgae and on the performance of algal extracts in terms of their antioxidant and antimicrobial properties. Overall, the characterization of algal extracts would contribute to the large-scale exploitation of these marine organisms as natural sources of bioactive compounds with health-enhancing properties, to be further employed in different sectors, including food, cosmetic, and pharmaceutical industries (Figure 1).

## 2. Materials and Methods

### 2.1. Chemicals and Reagents

All solvents were bought from Carlo Erba Reagents S.A. (Barcelona, Spain). Folin–Ciocalteu reagent, and 2,2-diphenyl-1-picrylhydrazyl (DPPH) were acquired from Sigma-Aldrich (Madrid, Spain). Gallic acid (GA), ascorbic acid (AA), Trolox (6-hydroxy-2,5,7,8-tetramethylchroman-2-carboxylic acid), ferric chloride, 2,4,6-Tri(2-pyridyl)-s-triazine (TPTZ), sodium acetate, dimethyl sulfoxide (DMSO), and lactic acid were from Sigma–Aldrich (Steinheim, Germany). The culture media Mueller–Hinton broth (MHB) and Mueller–Hinton II agar were acquired from Sigma-Aldrich (Madrid, Spain) and Liofilchem (Roseto degli Abruzzi, Italy), respectively.

### 2.2. Algae Sampling and Preparation

Brown algae samples were collected by Algamar (www.algamar.com (accessed on 18 July 2021)) from the Galician coast (NW Spain) in the winter season of 2019. Nine different brown algae species, namely: *Undaria pinnatifida*, *Himanthalia elongata*, *Bifurcaria bifurcata*, *Sargassum muticum*, *Laminaria ochroleuca*, *Saccharina latissima*, *Pelvetia canaliculata*, *Fucus spiralis*, and *Ascophyllum nodosum* were sorted, classified, and washed abundantly with tap water to remove salt, sand, and other debris. Afterward, samples were lyophilized (LyoAlfa10/15, Telstar, Thermo Fisher Scientific), pulverized into a fine powder by a blender, and stored at −20 °C until extraction.

### 2.3. Extraction Procedure

Algal samples were subjected to heat-assisted extraction The following solvents were selected for the study, based on their polarity characteristics (polarity index, PI): ethanol (EtOH, PI = 5.2); acetone (AcO, PI = 5.1); ethyl acetate (EtAc, PI = 4.4); chloroform (Chl, PI = 4.1); and hexane (Hex, PI = 0.1). For the extraction process [19], 0.6 g of alga were placed into a dark amber flask with 20 mL of solvent, and the mixture was stirred at 150 rpm using Thermo Scientific™ Cimarec™ Micro Stirrers for 24 h in a water bath at 50 °C. Afterward, the supernatant was collected and 10 mL of fresh solvent were added to the remaining algae and placed again in the orbital shaker for 1 h. This last step was repeated once and all the supernatants were collected. The resulting crude extract was then centrifuged to eliminate the remaining algae residues and supernatants were evaporated to dryness and further resuspended in 10 mL of ethanol:water (80:20, *v*:*v*) to obtain the algae extracts that will be employed in the different assays.

To figure out the extraction yield, 5 mL of algae extracts were deposited in crucibles (previously conditioned and weighted) and transferred to an oven at 104 °C for 24 h. Then, crucibles were placed in a desiccator until cooled down and weighted again. The yield (%) was calculated according to Equation (1), with *P*0 the initial weight, *P*1 the crucible weight, and *P*2 the weight after lyophilization.

(1) Yield (%)=[(P2−P1)/P0]×100

### 2.4. Total Phenolic Content Determination

The total phenolic content (TPC) of algae extracts was determined by a colorimetric assay, following an adaptation of the method developed by Singleton and Rossi [20]. A mixture of 75 µL of deionized water, 25 µL of Folin–Ciocalteu reagent (diluted 1:10) and 25 µL of algae extract was incubated in the dark for 6 min and then 100 µL of Na_2_CO_3_ (75 g/L) were added. This mixture was incubated for 90 min at room temperature in the dark. After incubation, the absorbance at 765 nm was measured and results were expressed as gallic acid equivalents (GAE) per g dry extract. Determinations were conducted in triplicate.

### 2.5. Antioxidant Activity Determination

The antioxidant activity of algae extracts was found colorimetric by two different assays, using a Synergy HT W/TRF Multi Mode Microplate Reader with Gen5 2.0 software (BioTek Instruments, Winooski, VT USA), was employed. All the experiments were conducted in triplicate.

#### 2.5.1. DPPH-Radical Scavenging Activity (DPPH-RSA) Assay

The DPPH (2,2-diphenyl-1-picrylhydrazyl)-RSA assay is based on the bleaching of this radical under the presence of antioxidant-containing extracts. Thus, the DPPH-RSA activity of algae extracts was measured spectrophotometrically at 517 nm, against the stable nitrogen radical DPPH. In this technique, 25 µL of algae extracts were mixed with 200 µL of fresh DPPH ethanolic solution (40 mg/L) and let stand for 30 min in the dark. Results were expressed as Trolox equivalent (TE) per g of the dry extract [21]. Determinations were conducted in triplicate.

#### 2.5.2. Ferric Reduction Activity Power (FRAP) Assay

FRAP assay is based on the reduction of the complex Fe^3+^-TPTZ to Fe^2+^-TPTZ under acidic conditions, generating a blue complex. FRAP measurements were performed by adding 20 μL of the algae sample to 180 μL of FRAP reagent solution (sodium acetate: TPTZ: Fe^3+^, 10:1:1, *v*:*v*:*v*). The mixture was incubated at 37 °C for 4 min for color development and the absorbance was measured at 593 nm. FRAP results were expressed as ascorbic acid equivalents (AAE) per g of the dry extract [21]. Determinations were conducted in triplicate.

### 2.6. Antibacterial Tests

#### 2.6.1. Microorganisms and Culture Conditions

The antimicrobial activity of algae extracts was assessed against the following Gram-positive bacterial strains: *Staphylococcus aureus* (ATCC 25923), *Staphylococcus epidermidis* (NCTC 11047), and *Bacillus cereus* (ATCC 14579); and the Gram-negative strains: *Pseudomonas aeruginosa* ATCC 10145, *Salmonella enteritidis* (ATCC 13676), and *Escherichia coli* (NCTC 9001). Bacterial strains were stored at −80 °C in glycerol:water (15:75, *v*:*v*). Prior to the experimental runs, active cultures were grown in sterile Mueller–Hinton broth (MHB) at 37 °C overnight, and then an aliquot from each culture was transferred to fresh MHB and properly diluted to achieve the optical density of 0.09–0.11, measured at 600 nm [22], corresponding to the 0.5 MacFarland standard to reach 1–2 × 10^8^ colony formation units (CFU) for further assays.

#### 2.6.2. Agar Diffusion Assay

This essay was performed following the protocol adapted from the Clinical and Laboratory Standards Institute (CLSI) guidelines [22,23]. Briefly, 5 mL of algae extract were evaporated until dryness under a nitrogen flow at 25 °C to minimize oxidation. The remaining residue was then dissolved in 2 mL of DMSO and filter sterilized through 0.45-µm pore-size syringe filters. Later, 100 µL of such suspensions for every microorganism were seeded in Petri dishes containing Mueller–Hinton II agar and spread with sterile swabs. Plates were divided into three sections, including 15 µL of each test algae extract (test section), DMSO as (negative control section), and 40% lactic acid (positive control section). Once cultivated, plates were incubated at 37 °C for 24 h. Growth inhibition was quantified as inhibition circular zones, and their diameters were measured using a digital caliper rule. Triplicate plates were assessed for each microorganism.

### 2.7. Statistical Analysis

The experimental data were conducted by triplicate and expressed as the mean ± standard deviation (SD), and they were statistically analyzed through one-way ANOVA followed by Tukey-HSD *post-hoc* test. Significance level was adjusted at α = 0.05. The software used was STATISTICA v. 12 (StatSoft Inc., 2014, Street Tulsa, OK, USA).

## 3. Results and Discussion

### 3.1. Extraction Efficiency, Phenolic Content, and Antioxidant Activity

Recently, brown algae have gained great attention due to their richness in bioactive compounds with associated antioxidant and antimicrobial activities [24]. The results of extraction efficiency, in terms of extraction yield (%), total phenolic content (TPC), and antioxidant activity, by DPPH-radical scavenging activity (DPPH-RSA) and ferric-reduction antioxidant power (FRAP), of the nine macroalgae analyzed in this work are presented in Table 1.

Concerning extraction yield, in general, ethanolic extracts promoted the highest percentages for all algal species, showing values ranging 14.6–38.8%, with *Undaria pinnatifida* extracts showing the highest values (Table 1). This could be attributed to the high polarity associated with ethanol, being able to promote a non-selective extraction of different macroalgal components, proteins, and polysaccharides, thus showing higher recovery rates. As can be seen, the extraction yield was strongly dependent on the chemical nature of the solvent, especially polarity. The yield of a chemical extraction depends on distinct factors, like the selected matrix, extraction technique, time, temperature, as well as the chemical nature of extracting solvents. Among them, the solvent plays a critical role in the extraction yield, as it should combine an enhanced solubility of the target compounds involved in the extraction and the suitability to be applied to different matrices [24].

With respect to total phenolic content, the Folin–Ciocalteu method has been extensively applied to find TPC in macroalgae. Nevertheless, this determination should be always considered as an indicative value, instead of an accurate measure of phenolic compounds concentration, since possible interferences may occur with other antioxidant constituents, such as reducing sugars and some amino acids, that can overestimate the determined values [25]. According to the data presented in Table 1, both the solvent used for extraction and algal species played a significant effect on TPC (*p* < 0.05). Results ranged between 2.07 mg of GAE/g of dry extract, for *P. canaliculata* extracted with Hex, to 211.83 mg of GAE/g of dry extract, for *A. nodosum* extracted with EtAc.

Concerning the solvent, AcO enabled obtaining the highest TPC rates for *U. pinnatifida*, *H. elongata*, *P. canaliculata*, *S. latissima*, *B. bifurcata*, and *F. spiralis*, while EtAc promoted the highest values for *L. ochroleuca*, *S. muticum*, and *A. nodosum*. Regarding the species, *A. nodosum* extracts showed the highest TPC values (6–211 mg GAE/g dry extract) when using all solvents with exception of hexane, in which *B. bifurcata* showed the highest rates (Table 1). On the other hand, *S. latissima* showed the lowest TPC values overall, ranging from 2.4 to 16 mg GAE/g dry extract. TPC has been evaluated in earlier scientific studies. However, the results of the present study are not directly comparable, due to the differences in the extraction techniques, solvents and standard compounds used for the determination of equivalents. In addition, the chemical composition of macroalgae, and thus the TPC, is affected by several factors, including biological factors, such as species and age, and environmental factors, like geographical location and seasonal variations [25,26]. Therefore, great variations could be observed between studies [27]. For example, in the study by Sánchez-Carmargo et al. [28], TPC values of EtOH extracts from *S. muticum* obtained with pressurized liquid extraction were 93 mg GAE/g dry extract, while in the present work, the TPC value was 8.31 mg GAE/g extract, which was obtained by maceration. Similarly, Otero et al. [29] employed the same technique to extract phenolic compounds from *L. ochroleuca* and reported TPC values of 6 and 83 mg GAE/g in Hex and EtOH dry extracts, respectively. These TPC values were higher than those obtained in this study, which were 3.78 mg GAE/g for Hex and 2.81 mg GAE/g for EtOH *L. ochroleuca* dry extracts (Table 1). These differences could be attributed to the different extraction techniques. Thus, the comparison of our results with earlier studies provide limited information. On the other hand, it was found that solvent effectiveness to extract phenolic compounds from brown macroalgae followed the trend: (EtAc, AcO) > (ChL, Hex, EtOH), which coincides with previous studies [30]. In addition, no correlation was found (data not shown) between yield and TPC values, which agrees with earlier reported results [31].

Moreover, compared with vegetable sources, our results are in line with TPC values of agriculture-derived products as it is the case of tomato water extracts (12.15 ± 0.83 mg GAE/g) and acetonic asparagus extracts (113.65 ± 17.73 mg GAE/g) [32], for example. Such evidence opens a promising perspective to consider algae as potent sources of high-value bioactive molecules, being comparable with the widely exploited agricultural sources.

Due to the heterogeneous nature of antioxidant activity, simultaneous determinations are needed to characterize the different mechanisms of action involved in this bioactivity. Therefore, in this work antioxidant activity was determined by two methods, DPPH-RSA and FRAP assays [21]. DPPH-RSA is a technique based on the reduction of the DPPH radical in the presence of a hydrogen-donating antioxidant. DPPH-RSA results are presented in Table 1, which ranged between 0.5 to 145.61 mg TE/g dry extract for AcO extract and Hex extract of *B. bifurcata*, respectively. In contrast, it is important to note that no antioxidant activity, in terms of RSA, was reported for the EtOH extracts of *U. pinnatifida*, *H. elongata*, and *P. canaliculata*.

As it occurred for TPC, both algal species and solvent played a critical role on DPPH-RSA, as statistically proven (*p* < 0.05). Regarding species, the highest DPPH-RSA values were seen for the *B. bifurcata* extracts, when using all solvents but AcO, in which A. nodosum extracts showed the highest rates (Table 1). On the contrary, the lowest values were assessed in *P. canaliculata* (2.46–16.06 mg TE/g dry extract) and *S. muticum* extracts (4.01–16.19 mg TE/g dry extract). Concerning the solvent, EtAc was reported to be the most efficient solvent in terms of RSA for the species *U. pinnatifida*, *P. canaliculata*, *S. latissima*, *L. ochroleuca*, *S. muticum*, and *A. nodosum*, presenting values of 46.55, 16.06, 24.87, 72.73, 16.19, and 89.8 mg TE/ g dry extract, respectively, meanwhile hexane promoted the highest RSA values for *H. elongata* and *B. bifurcata*. Contrarily, EtOH extracts showed the lowest RSA values for most species (Table 1). As a result, no correlation was found between TPC and DPPH-RSA values, which agrees with the results reported previously [31]. This lack of correlation seems to imply that other compounds like sugars, amino acids, and pigments are also present on the crude extract obtained [33], this alludes to the Folin–Ciocalteu reaction, that has it was mentioned previously, overestimates the TPC value. This may justify the inconsistency between extraction yields and TPC in the algal samples.

The FRAP assay also assesses the antioxidant activity by measuring the potential to reduce the yellow ferric-TPTZ complex to a blue ferrous-TPTZ complex by electro-donating substances under acidic conditions [25]. Results for FRAP assay are presented in Table 1, which ranged from 0.87 (*P. canaliculata* extracted with Hex) to 79.97 mg AAE/g dry extract (*A. nodusum* extracted with EtAc). FRAP was also significantly affected by algal species and the nature of the extracting solvent. In general, *A. nodosum* extracts promoted the highest FRAP rates, which are in accordance with the results for TPC, suggesting a cause-effect relationship between the concentration of phenolic compounds and antioxidant activity on this species. This fact could be partially explained by considering that antioxidant like polyphenols may be more effective reducing agents for ferric iron but not so efficient in scavenging DPPH free radicals [34]. With respect to solvent, AcO was shown to be the most effective solvent for the species *U. pinnatifida*, *H. elongata*, *P. canaliculata*, *S. latissima*, and *B. bifurcata*, while EtAc promoted the highest FRAP values for *L. ochroleuca*, *F. spiralis*, and *A. nodosum* (Table 1). These results suggest that the compounds responsible for the antioxidant activity of these species present a semi-polar nature. However, as previously reported for TPC, the results of the present study are not comparable to earlier scientific studies, due to the differences in the experimental conditions.

### 3.2. Antimicrobial Activity

Table 2 presents the diameter values corresponding to the inhibition zones obtained for the extracts of brown algae species assessed against several Gram-positive and Gram-negative bacteria. For this assay, the most common food-related microorganisms were selected, considering the possible use of these algae extracts as food additives. Additionally, two microorganisms known to cause opportunistic infections (*S. aureus* and *S. epidermidis*) were also assessed. The agar diffusion method is commonly used as a preliminary screening for the antimicrobial activity of biological extracts. Nevertheless, there are different approaches to perform the qualitative analysis of the agar diffusion test [35]. Several authors used different evaluation criteria [36,37]. In this study, it was used the classification proposed by CLSI. According to this classification, the diameter of the inhibition growth zone measured allows the classification of bacteria as susceptible (S, ≥20 mm), intermediate (I, 15–19 mm), or resistant (R, ≤14 mm) [23].

**Figure 2:**
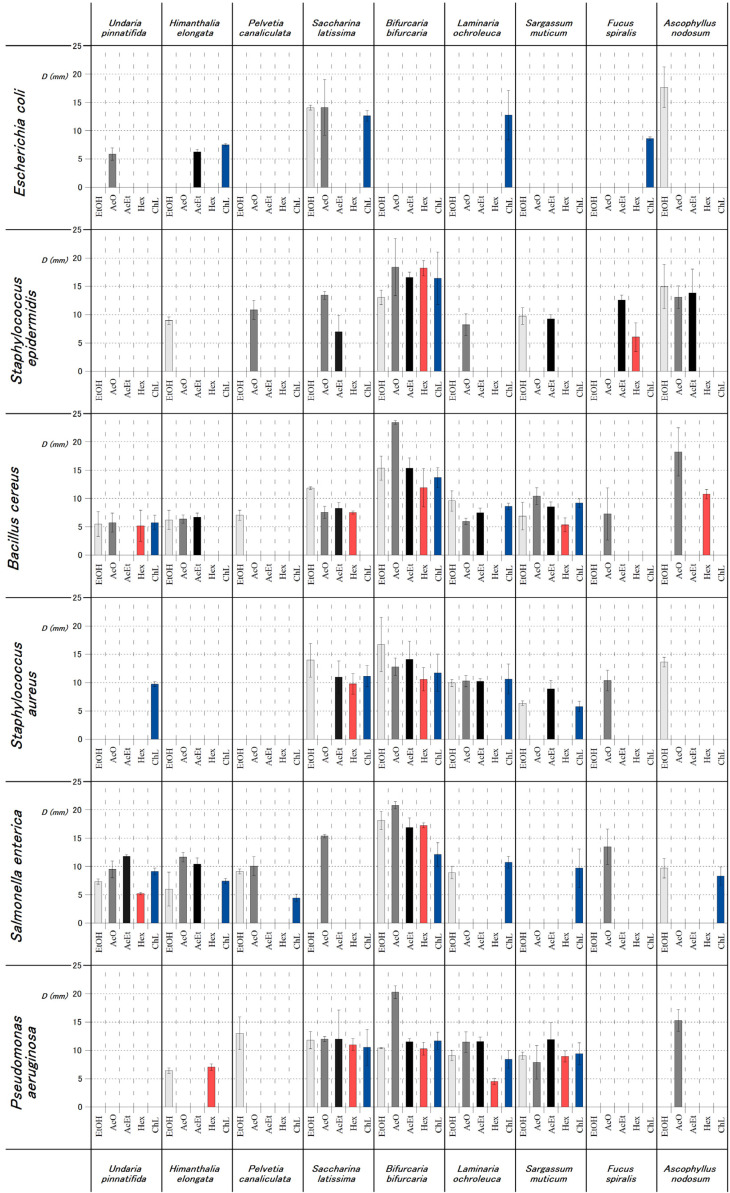
Diameter values (D, mm) for the inhibition growth values reported for the antimicrobial activity of brown algae extracts. Vertical bars show standard deviation of three replicates.

In fact, the growth inhibition of all microorganisms included in this study was highly dependent on the algal species and the solvent used for extraction. It can be observed that four macroalgae showed significant antimicrobial potential: *B. bifurcata*, *S. latissima*, *L ochroleuca*, and *S. muticum*. According to the results, *B. bifurcata* contributed to a total of 25% of the positive inhibitory responses, followed by *S. latissima* (16%), *L. ochroleuca* (14%), and *S. muticum* (13%). Notwithstanding, *A. nodosum* extracts only showed inhibition in a reduced number of cases but with remarkable inhibition zones produced, since they have an average diameter of 13.55 mm, only surpassed by *B. bifurcata* performance (Table 2). On the other hand, *P. canaliculata* was the algae with the weakest inhibitory responses. The remaining algae species handled 35% of the inhibition halos obtained. Generally, *B. bifurcata* extracts presented the most promising antimicrobial results among the nine algae species studied. The tested *B. bifurcata* extracts presented a significant activity, being able to inhibit the growth of all the tested bacteria except for *E. coli* (Figure 2), with AcO extract showing the greatest inhibition zones. Using this extract, the most susceptible bacteria were *B. cereus* (23.43 mm), *S. enteritidis* (20.84 mm), and *P. aeruginosa* (20.29 mm) (Table 2), while solvent did not play a significant role in the inhibition of *S. aureus* and *S. epidermidis*.

Our results also clarifies that the most resistant bacteria assessed was *E. coli*, being sensitive to a reduced number of extracts, while the most susceptible strains were *B. cereus*, *P. aeruginosa* and *S. enteritidis*. Considering the solvent, AcO significantly affected the antimicrobial activity of *B. bifurcata* extracts against *B. cereus*, *S. enteritidis* and *P. aeruginosa*, while in the rest of cases solvent did not play a critical role from a general point of view (Table 2). The results for *B. bifurcata* AcO extracts suggest that the observed antimicrobial effects may be associated with the higher recovery of phenolic compounds (Table 1). In the same way, the obtained data show that polar solvents were more efficient in the extraction of antimicrobial compounds from brown algae, since AcO extracts lead to 50% of the positive inhibition responses. However, it is also of major importance to consider the toxicity of the solvents employed: although acetone is one of the least toxic solvents, its vapors can cause temporary narcosis and eye irritation [38], whereas ethanol is recognized as a prominent solvent of importance for the food industry [39]. Furthermore, ethanol was the solvent that had the biggest extraction yield, so it should be considered as a preferential solvent. In this sense, our overall results were reinforced by the observations reported in previous studies, indicating that the antimicrobial capacity of algae extracts is solvent-dependent [40]. Consequently, this parameter should be optimized for different applications. Overall, the results obtained for algal extracts in terms of antimicrobial activity reinforces the hypothesis that they can be considered as promising sources of bioactive compounds of natural origin against foodborne microorganisms.

## 4. Conclusions

Brown algae are a source of interesting natural bioactive compounds that could be employed in the development of new industrial applications. However, the bioactive compound content in algal extracts is highly dependent on the extraction method applied. Our research was focused on the effect of five different extracting solvents: EtOH, AcO, EtAc, Chl, and Hex, on the extraction yield, polyphenolic content, and antioxidant and antimicrobial activities of nine brown algae from the NW coastal region of the Iberian Peninsula. As expected, all parameters were significantly affected by the species and the nature of the extracting solvent. In fact, the highest extraction yield was achieved when ethanol was used as solvent. Nevertheless, regarding TPC, DPPH-RSA, and FRAP values, EtAc and AcO were reported to be the most effective solvents, depending on the algae assessed, which suggests a differential composition of brown macroalgae in terms of phenolic compounds with antioxidant activity. Concerning the extracts, the highest TPC and FRAP values were found for *Ascophyllum nodosum* using EtAc as solvent, showing a possible correlation between polyphenol production and antioxidant activity in terms of reducing power, while the highest DPPH-RSA values were achieved by *Bifurcaria bifurcata* extracted with hexane, thus suggesting the hydrophobic nature of the antioxidant produced by this species.

On the other hand, the antimicrobial activity of algal extracts was also reported, also showing a strong dependence on the species and the nature of the extracting solvent, reinforcing the idea that the extraction of bioactive compounds should be individually optimized. In general, *Bifurcaria bifurcata* extracts showed the highest rates of antimicrobial activity against the whole panel of bacteria evaluated in this study, except for *E. coli*. Moreover, the use of AcO as solvent promoted a significant increase in the inhibitory effectiveness against *B. cereus*, *S. enteritidis*, and *P. aeruginosa*, thus proposing that *B. bifurcata* should be considered as a powerful candidate for its large-scale application by industrial sectors. Furthermore, considering the screening results, different strategies should be explored in the next future to the most significantly performing algae, including the optimization of the extraction parameters and the application of different extraction techniques such as ultrasound- and microwave-assisted extraction.

## Figures and Tables

**Table 1:** Extraction yield, TPC, DPPH-RSA, and FRAP values of nine brown algae species extracts using five different solvents ^1^.

Species	Solvent	Extraction Yield (%)	TPC	DPPH-RSA	FRAP
			mg GAE/g Dry E	mg TE/g Dry E	mg AAE/g Dry E
***Undaria pinnatifida***	EtOH	38.8	3.68 ± 0.35 efD	-±-	1.50 ± 0.02 cD
	AcO	3.40	41.5 ± 3.95 dA	13.79 ± 0.92 dC	15.89 ± 1.23 eA
	EtAc	2.40	16.53 ± 1.46 fB	46.55 ± 0.97 cA	9.51 ± 0.62 fgB
	Hex	2.20	3.46 ± 0.30 dD	33.92 ± 2.86 dB	1.78 ± 0.17 gD
	ChL	3.20	10.29 ± 1.07 deC	36.32 ± 1.71 cB	8.05 ± 0.47 bC
***Himanthalia elongata***	EtOH	27.0	30.26 ± 2.28 dC	-±	10.26 ± 1.83 bC
	AcO	3.6	162.22 ± 5.98 bA	5.19 ± 0.51 eD	62.98 ± 2.27 aA
	EtAc	0.20	53.34 ± 4.45 cB	54.24 ± 3.26 cB	28.12 ± 2.45 cB
	Hex	2.10	6.60 ± 0.22 cD	75.33 ± 8.52 bA	2.76 ± 0.10 deD
	ChL	3.20	13.07 ± 0.45 cdD	16.52 ± 2.09 dC	4.87 ± 0.58 deD
***Pelvetia canaliculata***	EtOH	15.6	49.49 ± 3.32 cB	-±	12.88 ± 0.71 bB
	AcO	12.2	87.32 ± 2.90 cA	2.46 ± 0.16 efD	21.14 ± 0.52 dA
	EtAc	6.90	35.61 ± 3.25 deC	16.06 ± 0.84 eA	13.07 ± 0.20 efB
	Hex	7.60	2.07 ± 0.17 eD	5.98 ± 0.57 efC	0.87 ± 0.08 hD
	ChL	7.7	7.66 ± 0.43 efD	7.58 ± 0.79 deB	5.39 ± 0.29 dC
***Saccharina latissima***	EtOH	18.0	2.44 ± 0.17 fD	1.23 ± 0.02 dE	1.97 ± 0.03 cD
	AcO	2.90	16.53 ± 0.63 eA	21.59 ± 1.8 cB	8.56 ± 0.27 fA
	EtAc	1.40	12.36 ± 0.89 fB	24.87 ± 2.29 dA	6.88 ± 0.37 ghB
	Hex	1.70	3.69 ± 0.27 dD	12.59 ± 0.00 eC	2.48 ± 0.25 efCD
	ChL	0.60	7.36 ± 0.32 fC	5.97 ± 0.53 eD	3.02 ± 0.11 fC
***Bifurcaria bifurcata***	EtOH	24.1	11.39 ± 0.32 eC	73.54 ± 2.95 aC	2.76 ± 0.15 cB
	AcO	10.8	86.08 ± 5.54 cA	0.50 ± 0.03 fD	35.38 ± 2.16 cA
	EtAc	4.40	21.88 ± 0.94 efB	110.58 ± 5.09 aB	3.46 ± 0.28 hB
	Hex	1.60	18.82 ± 0.85 aB	145.61 ± 4.6 aA	3.60 ± 0.31 cB
	ChL	4.40	16.81 ± 0.77 bBC	107.28 ± 8.95 aB	2.86 ± 0.17 fB
***Laminaria ochroleuca***	EtOH	19.2	2.81 ± 0.25 fC	5.38 ± 0.18 dD	1.24 ± 0.70 cD
	AcO	0.80	14.59 ± 0.75 eB	22.83 ± 1.59 cC	11.86 ± 0.53 fB
	EtAc	1.40	32.46 ± 2.54 deA	72.73 ± 4.01 dA	18.33 ± 1.27 deA
	Hex	0.60	3.78 ± 0.24 dC	56.95 ± 3.09 dB	2.21 ± 0.14 fgD
	ChL	2.10	11.82 ± 1.01 dB	6.13 ± 0.13 eD	7.14 ± 0.56 bcC
***Sargassum muticum***	EtOH	17.9	8.31 ± 0.33 efC	4.01 ± 0.23 dD	4.18 ± 0.1 cB
	AcO	3.40	25.89 ± 2.54 eB	5.48 ± 0.21 eBC	17.46 ± 1.09 deA
	EtAc	0.30	41.63 ± 3.97 cdA	16.19 ± 1.08 eA	18.33 ± 1.51 dA
	Hex	2.00	10.38 ± 0.41 bC	6.06 ± 0.62 efB	5.99 ± 0.29 bB
	ChL	2.10	15.35 ± 0.97 bcC	4.65 ± 0.39 eCD	6.16 ± 0.65 cdB
***Fucus spiralis***	EtOH	14.6	95.75 ± 5.28 bC	60.34 ± 3.07 bA	46.27 ± 2.28 aB
	AcO	7.80	184.22 ± 12.82 aA	57.48 ± 2.08 bA	48.18 ± 1.00 bB
	EtAc	5.30	123.67 ± 4.50 bB	52.87 ± 3.14 cA	68.97 ± 4.54 bA
	Hex	5.70	10.91 ± 0.49 bD	56.35 ± 2.43 cA	3.00 ± 0.07 dC
	ChL	7.00	17.15 ± 1.24 bD	53.20 ± 3.86 bA	3.93 ± 0.27 efC
***Ascophyllum nodosum***	EtOH	18.2	117.2 ± 6.33 aC	52.52 ± 2.27 cC	43.69 ± 1.03 aC
	AcO	15.4	183.13 ± 5.30 aB	73.3 ± 2.26 aB	59.97 ± 2.03 aB
	EtAc	10.3	211.83 ± 18.22 aA	89.8 ± 4.28 bA	79.97 ± 1.11 aA
	Hex	8.00	6.18 ± 0.36 cD	3.76 ± 0.18 fE	8.09 ± 0.01 aE
	ChL	9.40	23.73 ± 0.80 aD	29.05 ± 1.26 cD	14.360.92 aD

^1^ Different lower-case letters show significant differences (*p* < 0.05) between algal extracts within the same solvent, while different capital letters indicate significant differences (*p* < 0.05) between solvents used for the same algal species.

**Table 2:** Average diameter of inhibition zone ± standard deviation (mm) of brown algae extracts positive and negative controls ^1^.

Species	Solvent	Inhibition Zone (mm)					
		*Gram* (+)			*Gram* (−)		
		*S. aureus*	*S. epidermidis*	*B. cereus*	*E. coli*	*S. enteritidis*	*P. aeruginosa*
***Undaria pinnatifida***	EtOH	-	-	5.49 ± 2.19 cA	-	7.34 ± 0.47 bcC	-
	AcO	-	-	5.74 ± 1.68 bA	5.86 ±1.08 b	9.51 ± 1.47 dB	-
	EtAc	9.75 ± 0.44 b	-	-	-	11.81 ± 0.30 bA	-
	Hex	-	-	5.19 ± 2.75 cA	-	5.21 ± 0.20 bD	-
	ChL	-	-	5.74 ± 1.30 cA	-	9.11 ± 0.60 abBC	-
***Himanthalia elongata***	EtOH	-	8.94 ± 0.67 b	6.20 ± 1.68 cA	-	5.65 ± 2.50 cC	6.42 ± 0.49 cA
	AcO	-	-	6.36 ± 0.71 bA	-	11.67 ± 0.85 bcdA	-
	EtAc	-	-	6.71 ± 0.71 bA	6.28 ± 0.42 A	10.42 ± 1.14 bAB	7.06 ± 0.58 bA
	Hex	-	-	-	-	-	-
	ChL	-	-	-	7.52 ± 0.22 bA	7.44 ± 0.46 bcBC	-
***Pelvetia canaliculata***	EtOH	-	-	7.05 ± 0.88 c	-	9.14 ± 0.42 bA	13.01 ± 2.90 a
	AcO	-	-	-	-	10.06 ± 1.65 cdA	-
	EtAc	-	10.84 ± 1.70 bc	-	-	-	-
	Hex	-	-	-	-	-	-
	ChL	-	-	-	-	4.43 ± 0.67 cB	-
***Saccharina latissima***	EtOH	13.96 ± 2.98 abA	-	11.83 ± 0.22 abA	14.08 ± 0.43 bA	-	11.83 ± 1.52 abA
	AcO	-	13.41 ± 0.69 abA	7.56 ± 1.06 bB	14.12 ± 4.92 aA	15.38 ± 0.31 b	12.00 ± 0.45 bA
	EtAc	10.98 ± 2.88 abA	6.98 ± 2.88 cB	8.28 ± 1.01 bB	-	-	11.98 ± 5.14 aA
	Hex	9.78 ± 1.83 aA	-	7.52 ± 0.28 bcB	-	-	10.99 ± 1.06 aA
	ChL	11.13 ± 1.86 aA	-	-	12.65 ± 0.94 aA	-	10.55 ± 3.18 aA
***Bifurcaria bifurcata***	EtOH	16.74 ± 4.79 aA	13.05 ± 1.28 abA	15.35 ± 2.13 aB	-	18.14 ± 1.60 aAB	10.43 ± 0.08 abB
	AcO	12.81 ± 1.56 aA	18.39 ± 5.03 aA	23.45 ± 0.35 aA	-	20.84 ± 0.63 aA	20.29 ± 1.13 aA
	EtAc	14.12 ± 3.22 aA	16.55 ± 0.95 aA	15.37 ± 1.79 aB	-	16.88 ± 1.71 aB	11.53 ± 0.55 abB
	Hex	10.60 ± 2.05 aA	18.22 ± 1.36 aA	11.90 ± 3.39 aB	-	17.28 ± 0.43 aAB	10.03 ± 1.13 abB
	ChL	11.73 ± 3.29 aA	16.44 ± 4.63 A	13.68 ± 1.74 aB	-	12.14 ± 2.07 aC	11.74 ± 1.49 aB
***Laminaria ochroleuca***	EtOH	9.94 ± 0.64 bcA	-	9.57 ± 1.81 bcA	-	8.93 ± 1.07 bcB	9.12 ± 0.90 bcA
	AcO	10.27 ± 0.99 bA	8.20 ± 1.95 b	5.95 ± 0.54 bB	-	-	11.49 ± 1.82 bcA
	EtAc	10.24 ± 0.47 abA	-	7.48 ± 0.85 bAB	-	-	11.55 ± 0.81 abA
	Hex	-	-	-	-	-	4.53 ± 0.55 cB
	ChL	10.64 ± 2.65 aA	-	8.61 ± 0.56 bA	12.76 ± 4.38 a	10.75 ± 1.05 abA	8.43 ± 1.58 aA
***Sargassum muticum***	EtOH	6.36 ± 0.41 cB	9.73 ± 1.48 bA	6.89 ± 2.44 cAB	-	-	9.06 ± 0.64 bcA
	AcO	-	-	10.42 ± 1.50 bA	-	-	7.89 ± 2.94 cA
	EtAc	8.90 ± 1.50 bA	9.22 ± 0.77 bcA	8.53 ± 0.89 bAB	-	-	11.89 ± 3.04 aA
	Hex	-	-	5.35 ± 1.22 cB	-	-	8.94 ± 0.97 bA
	ChL	5.76 ± 0.91 bA	-	9.19 ± 0.78 bA	-	9.71 ± 3.38 ab	9.39 ± 1.95 aA
***Fucus spiralis***	EtOH	-	-	-	-	-	-
	AcO	10.38 ± 1.81 b	-	7.28 ± 4.60 b	-	13.49 ± 3.12 bc	-
	EtAc	-	12.57 ± 0.86 abA	-	-	-	-
	Hex	-	6.04 ± 2.52 bB	-	-	-	-
	ChL	-	-	-	8.60 ± 0.29 b	-	-
***Ascophyllum nodosum***	EtOH	13.63 ± 0.85 ab	14.97 ± 3.92 aA	-	17.70 ± 3.58 a	9.71 ± 1.72 bA	-
	AcO	-	13.07 ± 2.01 abA	18.24 ± 4.26 aA	-	-	15.28 ± 1.91 b
	EtAc	-	13.85 ± 4.21 abA	-	-	-	-
	Hex	-	-	10.79 ± 0.84 abB	-	-	-
	ChL	-	-	-	-	8.30 ± 1.61 bA	-
**DMSO**		-	-	-	-	-	-
**Lactic acid**		18.55 ± 3.75	17.20 ± 3.83	16.75 ± 2.98	18.52 ± 3.63	19.19 ± 3.23	18.70 ± 2.64

^1^ Different lower-case letters indicate significant differences (*p* < 0.05) between algal extracts within the same solvent for each microorganism, whereas different capital letters indicate significant differences (*p* < 0.05) between solvents used for the same algal species for each microorganism.

## Data Availability

The authors do not have anything to declare.

## Abbreviations

AAE	Ascorbic acid equivalents
AcO	Acetone
ATCC	American type culture collection
CFU	Colony formation units
ChL	Chloroform
DMSO	Dimethyl Sulfoxide
DPPH	1,1-diphenyl-2-picryl hydrazyl
DW	Dry weight
EtAc	Ethyl acetate
EtOH	Ethanol
FRAP	Ferric reduction activity power
FW	Fresh weight
GAE	Gallic acid equivalents
Hex	Hexane
MHB	Mueller–Hinton broth
PI	Polarity index
TPC	Total phenolic content
TPTZ	Fe^3+^-2,4,6-tri(2-pyridyl)-S-triazine
Trolox	6-hydroxy-2,5,7,8-tetramethylchroman-2-carboxylic acid
